# Exploring Chinese EFL teachers' knowledge and beliefs relating to the teaching of English reading in public primary schools in China

**DOI:** 10.1002/dys.1630

**Published:** 2019-08-15

**Authors:** Meina Luo, Susan Main, Graeme Lock, R. Malatesha Joshi, Chenyin Zhong

**Affiliations:** ^1^ College of Foreign Languages Zhejiang Normal University Jinhua Zhejiang Province China; ^2^ School of Education Edith Cowan University Joondalup Western Australia Australia; ^3^ College of Education and Human Development Texas A&M University College Station Texas USA; ^4^ College of Teacher Education Zhejiang Normal University Jinhua Zhejiang Province China

**Keywords:** a skills perspective, English reading instruction, explicit instruction, teacher education, teachers' beliefs, teachers' knowledge

## Abstract

The present study explored knowledge and beliefs about reading instruction of Chinese teachers teaching English as a foreign language (EFL). Theoretical Orientation to Reading Profile and the Survey of Basic Language Constructs Related to Literacy Acquisition were administered to 262 EFL teachers in the south‐eastern part of China. Additionally, three teachers were interviewed, and their instructional practices were observed. The results showed that there was no correlation between teachers' self‐efficacy beliefs and the performance on the knowledge of basic language construct survey. However, it was found that teachers' knowledge, beliefs, and instructional practices were mediated by the Chinese EFL contextual factors. Educational and practical implications are discussed.

## 1 INTRODUCTION

There are more than 390 million people learning English as a foreign language (EFL) in Mainland China (Wei & Su, 2012). Thus, there is an emphasis in English teaching in China. To meet the needs of the population learning English in China, the English curriculum standards have shifted towards cultivating core competencies in students' English language to incorporate a more constructivist approach than was used previously. This has posed challenges for Chinese EFL teachers because it is necessary for them to hold the requisite knowledge and skills themselves before they can cultivate students' core competencies. English reading instruction plays an important role in facilitating students' acquisition of these core competencies, as claimed by Wang (2017), because it can enhance students' skills in critical thinking (Koek, Janssen, Hakemulder, & Rijlaarsdam, 2016), building linguistic confidence, and fostering language‐learning abilities (Huang, 2014). There is a need to ensure that Chinese EFL teachers have the knowledge and skills to align with the curriculum changes and evidence‐based teaching practices that support reading development. A wealth of literature has documented that teachers' knowledge and beliefs influence their instructional practices (Basturkmen, 2012; Farrell & Bennis, 2013; Johnson, 1992; Main, 2014; McCutchen, Stull, Herrera, Lotas, & Evans, 2014; Piasta, Connor, Fishman, & Morrison, 2009; Shulman, 1986; Si, 2013; Westwood, Knight, & Redden, 2005). However, few studies have probed into EFL teachers' knowledge and beliefs in relation to reading instruction in China. Therefore, the purpose of this study was to explore Chinese primary school EFL teachers' knowledge and beliefs concerning effective teaching of English reading in the Chinese context.

To accommodate the demands of economic and technological development, as China is expanding its role in global leadership, as well as addressing various problems occurring at different levels of English education, the English curriculum standards in China formulated in 2001 were renewed in 2011. The 2011 version adopts a futuristic perspective in line with world trends and has several distinctive features (Ministry of Education, 2011). First, English education is tied to the cultivation of students' comprehensive humanistic competencies rather than just acquiring linguistic knowledge and skills (Liu, 2011). Second, a strong communication‐oriented pedagogy with task‐based language teaching is highly recommended.

The English curriculum reform initiative has promoted a shift towards developing students' English core competencies that are composed of linguistic competencies, learning abilities, thinking qualities, and cultural character (Wang, 2016). What is noticeable is the crucial role of reading instruction that plays in cultivating these competencies. To enhance reading instruction, the Chinese Primary and Middle School Students' English Reading Literacy Framework has been formulated (Wang & Chen, 2016). This framework encompasses two major parts: reading competencies and reading character. Reading competencies comprise decoding ability, linguistic knowledge, reading comprehension, and cultural awareness. Reading character comprises reading habits and reading experiences. Reading competencies and reading character complement each other—Reading competencies are the foundation of a reading character, and a reading character provides sustained and powerful support for reading competencies. Thus, it is essential that teachers cultivate students' reading competencies before reading character can be established. Interestingly, Hempenstall (2016) also highlighted similar qualities for reading instruction of native speakers of English (L1). The formulation of the above framework in China indicates that early English reading education is being devoted more attention yet also presents tremendous challenges for primary school English teachers concerning whether they have adequate knowledge and skills to foster students' reading development.

Although the debate about the best way to teach reading continues, more researchers and teachers have begun to endorse a balanced approach drawing on strengths of both code‐emphasis and meaning‐emphasis approaches (Gibbons, 2002; Pressley & Allington, 2014). Yet it is difficult to define what a balanced approach is and how balance is created as advocates of different approaches have different beliefs about how balance is achieved (Lyon, 1997; Pressley & Allington, 2014). However, findings of major large‐scale studies of reading (Australian Government Department of Education Science and Training, 2005; National Reading Panel, 2000; Rose, 2006) and a number of recent studies (Hempenstall, 2016; Rupley, Blair, & Nichols, 2009) have clearly shown that explicit instruction of the fundamental components of reading is the most effective teaching method for beginning reading.

The National Reading Panel (2000) in the United States reviewed more than 100,000 studies that were based on sound research methodology about effective approaches for teaching children to read and found five essential areas for effective teaching of English reading: phonemic awareness, phonics, fluency, vocabulary, and comprehension. The findings also indicated that explicit and systematic teaching of phonemic awareness and phonics resulted in the improvement of children's early reading and spelling abilities. However, Dickinson, Golinkoff, and Hirsh‐Pasek (2010) cautioned against ignoring the significance of oral language development when teaching reading. Konza (2010) also asserted the importance of oral language in the acquisition of reading and included this as a sixth component to the five identified by the National Reading Panel. The “big six” was synthesized from major reviews of effective English reading instruction, and Konza asserted that their explicit and systematic teaching would be beneficial for most children. The development of oral language not only has been identified as an important precursor to phonological awareness but also has been noted as an ongoing process that contributes to proficiency in subsequent reading skills. Further, Blair, Rupley, and Nichols (2007) contended that teachers play a major role in effective reading instruction and concluded that besides the common instructional features, effective reading teachers believe in their teaching abilities and expect students to be successful. Therefore, exploring teachers' knowledge and beliefs about teaching and learning English reading is important to understand their classroom instructional practices.

### 1.1 Impact of teachers' knowledge

Carter (1990) proposed that teachers' knowledge is the knowledge that teachers have at their disposal at a particular moment, which underlies their actions. A substantial body of literature has documented the significant impact teachers' knowledge has on their instructional practices and students' learning outcomes (McCutchen et al., 2014; Piasta et al., 2009; Shulman, 1986). A study by Piasta et al. (2009) examined the relationship between teachers' reading‐related knowledge in English, teachers' reading instructional practices, and students' reading gains and found an interaction between teachers' knowledge and decoding instruction in their instructional practices. Teachers with more reading‐related knowledge provided more explicit instruction that was more effective in improving students' word‐reading skills. In contrast, less knowledgeable teachers, even though they spent more time in explicit reading instruction, were associated with weaker student word‐reading gains. Consequently, Piasta et al. called for developing a specialized teaching body of knowledge about reading to inform effective reading instruction.

Although such research indicates the impact of using basic language knowledge to improve classroom practices and student outcomes, particularly in reading, other research indicates significant insufficiencies in such knowledge among many teachers. A seminal study (Moats, 1994) investigated experienced teachers' knowledge of basic language concepts (such as phonemes, morphemes, and phonics) through a survey. The results showed that, overall, the teachers held an inadequate knowledge base for teaching reading. For example, they were unable to distinguish between phonology, phonetics, and phonics. According to Moats (1994), the reason for teachers' poor performance could be a lack of explicit instruction of basic knowledge of English language structure in teacher education programmes; hence, its inclusion was strongly advocated. A recent research study by Zhao, Joshi, Dixon, and Huang (2015) examined Chinese primary school EFL teachers' knowledge and skills of basic language constructs through the Survey of Basic Language Constructs Related to Literacy Acquisition for Chinese EFL Teachers (SBLCRLA). The findings indicated that participant teachers were less able to display explicit knowledge of sublexical items, such as phonemes and morphemes, than implicit skills relating to certain language knowledge, such as counting syllables. The findings also revealed that teachers lacked usable knowledge about language for teaching reading, including concepts such as phonological and phonemic awareness. However, without data from classroom observation, it is difficult to judge whether there is a disconnection between teachers' reported language skills and the actual use of knowledge in the class. In spite of that, these previous findings did suggest the need to include explicit teaching of basic language concepts in teacher education programmes, based on the link between knowledge and practice in other studies.

### 1.2 Impact of teachers' beliefs

Teachers' beliefs are those related to “teachers' pedagogic beliefs, or those beliefs of relevance to an individual's teaching” (M. Borg, 2001, p. 187). Teachers' beliefs guide and inform the instructional practices by serving as a filter for interpretation of new phenomena (Pajares, 1992). Kagan (1992) claimed that beliefs “lie at the very heart of teaching” (p. 85), and de Lemos (2005) acknowledged the impact of teachers' beliefs on their practices while advocating that teachers should be informed about why certain approaches are effective.

Previous studies of language teachers' beliefs have focussed on the relationship between teachers' beliefs and their instructional practices, revealing mixed results that show a complex relationship between the two (Basturkmen, 2012; Farrell & Bennis, 2013). Johnson's (1992) study indicated that more than half of the ESL teachers held clear theoretical beliefs that consistently reflect one particular methodological approach to second language teaching and that teachers with clear theoretical beliefs aligned their instruction more with their theoretical orientation. Other studies have identified that great variations exist between teachers' beliefs and their instructional practices (Farrell & Lim, 2005; Graden, 1996; Kinzer, 1988). Many factors have been found to influence the degree of consistency between teachers' practices and beliefs. Situational constraints (S. Borg, 2003; Fang, 1996), teacher preparation (Deal & White, 2006), school context, and learners' academic and social background (Larenas, Hernández, & Navarrete, 2015) have been identified as influencing teachers' practices.

To date, research on teachers' beliefs in China mainly addresses three areas of concern: the identification of language teachers' belief systems (H. Chen, 2009), the influence of teachers' beliefs on their practices (Qin, 2007; Zheng, 2004), and the factors influencing teachers' beliefs (F. J. Zhang & Y. B. Liu, 2011). Only a few studies on teachers' beliefs have been conducted at the primary education level (Wang, 2007; L. C. Zhang, 2008). Using questionnaires, L. C. Zhang (2008) examined 97 primary school English teachers' beliefs about English language teaching. It is noticeable that in those teachers' beliefs, a solid professional knowledge base is among the essential characteristics of quality primary school English teachers.

In summary, the literature reviewed indicates that although research on teachers' theoretical orientation to reading and knowledge base for reading instruction has been richly documented in English L1 and L2 contexts, it is, however, still rare in the Chinese EFL context. In view of these gaps, this research aimed to explore Chinese primary school EFL teachers' knowledge and beliefs pertaining to English reading instruction. The research questions were formulated as follows:

1. What are Chinese EFL teachers' beliefs about the teaching of English reading in public primary schools in China?
2. How does Chinese EFL teachers' foundational knowledge of reading in English relate to their beliefs about teaching reading?
3. How do the case study teachers' knowledge and beliefs about teaching of English reading influence their instructional practices?

## 2 METHODOLOGY

### 2.1 Method

A mixed‐methods approach involving collecting data from multiple sources: surveys, interviews, and classroom observations, was employed. The research also sought to understand teachers' knowledge and beliefs through an in‐depth focus on specific cases. With this design, first, quantitative data of all participants were generated by surveys, and then, the researchers collected qualitative data from three case study participants through interviews and classroom observations. Following this, the data were analysed; data from different sources were converged for interpretation, and across‐method triangulation was used.

### 2.2 Participants

The survey participants were included based on the criterion that they were English teachers in public primary schools of Zhejiang Province, China. To obtain a larger sample for surveys, snowball sampling was also employed.

Two hundred and ninety teachers from different parts of Zhejiang Province were recruited to complete surveys during two teacher professional development (TPD) sessions held by Zhejiang Normal University (ZJNU) and during four sessions of TPD organized by local teacher training schools in Zhejiang. Two participants attending TPD sessions held by ZJNU contacted their colleagues, and 10 more participants were recruited using snowball sampling. Altogether, 300 paper‐and‐pencil questionnaires were administered, and 283 questionnaires were collected, of which 262 were valid.

Table 1 presents the demographic characteristics of the study population. As shown in Table 1, female teachers accounted for 93.1% (*N* = 244) of the participants, whereas there were only 18 male teachers, comprising 6.9% of the total sample. The uneven distribution of participants in terms of gender reflects the current composition of primary school English teachers in this area. This is also consistent with the findings of Wang (2007) and Wu and Yang (2008), who found that the English teaching profession comprises mostly female teachers in China. In addition, research on the influence of gender on students' achievement in learning ESL has provided ample evidence that female students outperform male students (Główka, 2014; Murphy, 2010) because they are more open to new linguistic forms in the target language (Ellis, 2008). This could explain why it is less likely for male students to be admitted into an English major programme in normal universities and to later become English teachers.

**Table 1:** Demographic characteristics of the study population

Variables	Levels	*F*	*P* (%)
Gender	Male	18	6.9
	Female	244	93.1
Educational background	English major	227	86.6
	Other majors	35	13.4
Educational institution	Teachers college and normal universities	198	75.6
	Other universities	64	24.4
School location	Cities	146	55.7
	Towns	78	29.8
	Rural areas	38	14.5
Highest qualification	College diploma	5	1.9
	Bachelor's degree	238	90.8
	Master's degree	15	5.7
	Other (less than 3‐year programme)	4	1.5
Years of teaching	1–3	36	13.7
	4–6	43	16.4
	7–18	146	55.7
	19–30	34	13
	30+	2	0.8
	Missing	1	0.4

In terms of educational background, most participants had studied English majors, comprising 86.6% of the total sample, whereas 35 participants had studied other majors, constituting 13.4% of the total sample. Further, 198 participants had graduated from teachers colleges and normal universities, comprising 75.6% of the sample, whereas 64 participants had graduated from other types of universities such as comprehensive universities and vocational technical institutes, comprising 24.4% of the sample. Regarding school locations, slightly more than half of the participants were from schools located in cities (55.7%), whereas 78 teachers were from schools located in towns (29.8% of the sample) and 38 teachers were from schools located in rural areas (14.5% of the sample), indicating the current rapid rate of urbanization in China.

Only a small number of teachers held a college diploma (*N* = 5) and other degrees such as the technical secondary school diploma that were programmes of less than 3‐year duration (*N* = 4), comprising 1.9% and 1.5% of the sample, respectively. The majority of teachers had obtained a bachelor's degree, comprising 90.8% of the sample. A minority of the participants held a master's degree (*N* = 15), comprising 5.7% of the sample. Thirty‐six participants (13.7%) had 1 to 3 years of teaching experience, whereas 16.4% of the participants (*N* = 43) were in their fourth to sixth years of teaching. Slightly more than half of the participants (*N* = 146) had 7 to 18 years of teaching experience, whereas 13% of the participants (*N* = 34) had 19 to 30 years of teaching experience, and two participants had more than 30 years of teaching experience.

In summary, in terms of the number of teachers, regions, gender, educational attainment, and teaching experience, the survey participants were representative of the overall population of the public primary school English teachers in Zhejiang Province, China.

The research also used purposeful sampling to select cases for in‐depth study. Potential participants were located according to the following criteria: (a) teachers come from public primary schools in Zhejiang Province; (b) teachers teach the upper grades, that is, grade 5 or grade 6 (in the Chinese educational system, primary schools range from grade 1 to grade 6 and admit children aged from 7 to 12 years old) because reading is taught separately in upper grades, whereas, in lower grades, teaching of English reading is integrated into teaching of other linguistic skills. The participants were recruited during a TPD session held by ZJNU. Three Chinese EFL teachers, Xiaomei, Qingqing, and Lili (pseudonyms), from two public primary schools of Zhejiang Province self‐selected as the participants and met the research criteria. More information about these teachers is provided later.

### 2.3 Data collection and analysis

Data were collected using multiple techniques: surveys, interviews, and classroom observations.

#### 2.3.1 Surveys

The measures included a short demographic questionnaire, the DeFord (1985) Theoretical Orientation to Reading Profile (TORP), and the SBLCRLA (Zhao et al., 2015). A short demographic questionnaire enabled collection of the teachers' personal information for comparison with patterns in the responses. The information collected included gender, educational background, the educational institutions from which the teachers had graduated, the teachers' school location, the teachers' highest qualification obtained, and the teachers' years of teaching experience.

The TORP consists of 28 items reflecting practices and beliefs about English reading instruction. DeFord (1985) validated the TORP instrument, and the validation process suggested that the TORP instrument is reliable (a Cronbach's *α* of .98) and valid. It has also been identified as a valid instrument to identify teachers' theoretical orientation to teaching reading by other researchers (Bos, Mather, Dickson, Podhajski, & Chard, 2001; McCutchen et al., 2002). Therefore, this instrument was used to uncover EFL teachers' beliefs about how reading is taught and learnt and to provide insight into the implicitly embedded beliefs and instructional practices of these teachers. The TORP uses a Likert scale response system, ranging from 1 to 5, with items classified into three categories: phonics, skills, and whole language. These categories reflect the three current conceptualizations of teaching reading: a decoding perspective (a phonics approach), a skills perspective (a balanced approach), and a whole‐language perspective (a whole‐language approach). The score is a general indicator of the respondent's orientation.

To provide additional information to understand Chinese EFL teachers' selection of teaching approaches, this research employed the SBLCRLA, originally developed by Joshi et al. (2009) and validated by Binks‐Cantrell, Joshi, and Washburn (2012) with high reliability (a Cronbach's *α* of .90). The survey comprises 46 items that evaluate participants' self‐perception, knowledge, and skills in basic language constructs. The current study's adapted version comprised 27 items, with some items removed from the original survey because of translation problems and unfamiliar terminology for Chinese teachers. The survey was administered to 630 in‐service teachers (Zhao et al., 2015). The items of the survey were grouped into two categories: knowledge and skills. Thus, the survey aimed to explore EFL teachers' phonemic, phonological, phonics, and morphological knowledge and skills. The surveys took approximately 40 min to complete. The participants in this study were asked to provide a personal code for the purpose of identifying the case study teachers. These codes enabled the researchers to isolate the case study teachers' surveys.

#### 2.3.2 Interviews with case study teachers

Interviewing provided opportunities to learn what teachers perceive, how they interpret their perceptions, and how such perceptions affect their classroom decision making and practices. Initial in‐depth interviews were conducted in the schools of case study teachers prior to classroom observations, and post‐observation interviews were held soon after the classroom observations. The semi‐structured Initial Observation Interview Guide (see Appendix A) was used to guide the initial observation interviews, which was open‐ended and primarily focussed on topics related to the participants' beliefs about effective reading instruction and their instructional practices. The post‐observation interview was more structured. The Post‐observation Interview Guide (see Appendix B) was modified after the classroom observations and varied among the participants. Both the initial and post‐observation interviews took approximately 40 min, and an audio‐recorder was used during the interviews, so the responses could be transcribed and analysed.

#### 2.3.3 Classroom observations of case study teachers

Classroom observations were employed to assist with validation of the findings obtained from the surveys and interviews. These observations could also implicitly reveal beliefs about reading instruction that the teachers did not mention during the interviews. Data collected from the observations also served as a reference point for the post‐observation interviews. The Reading Practices Guide—a modified version of the Literary Practices Guide (Konza, 2012) and renamed with the author's permission—was used. This guide, modified based on the type of reading environment in the Chinese EFL context and linked to the current English curriculum standards in China, provided information about the classroom environment, instructional activities, materials, and language choices in the classroom. In addition to audio‐recording the teachers' classroom instruction, information from the classroom observations was recorded as written field notes throughout the observation periods. Between four and six classes (40 min per class) for each participant teacher were observed.

Statistical Package for the Social Sciences software (V. 20) was used to analyse the data collected from the surveys. Descriptive statistics were generated, and, where appropriate, inferential statistics were used, including chi‐square tests and Mann–Whitney tests. Data from interviews and classroom observations were coded and analysed using a constant comparative method (Merriam, 2009). The research adopted a within‐case analysis using content reduction techniques, as well as cross‐case analysis, which helped identify the similarities and differences in teachers' beliefs and instructional practices.

## 3 RESULTS AND DISCUSSION

### 3.1 Teachers' beliefs about English reading instruction

The DeFord (1985) TORP was employed to identify the participants' theoretical orientation to teaching reading. The results indicated that 78% (*N* = 204) of the total 262 participants identified their theoretical orientation to reading instruction as skills based, whereas 22% (*N* = 58) held a decoding perspective, and none held a whole‐language perspective.

The ranking for the 28 items in order of preference is shown in Tables 2 and 3. The top five items were 3, 4, 21, 12, and 7 as indicated in Table 2, whereas the last five items were 13, 27, 10, 23, and 15 as shown in Table 3. The items with which all participants agreed were Items 3, 4, and 21, indicating that all participants agreed that, when encountering new words, decoding words is helpful to teach reading, good comprehension is linked with fluency and expression, and formal education in reading is essential to develop reading abilities. All participants disagreed with Item 15, suggesting that, when encountering a new word, guessing the meaning is unlikely to be encouraged.

**Table 2:** Means of the top five items of the Theoretical Orientation to Reading Profile

Ranking	Item	Content	Mean
1	3	Dividing words into syllables according to rules is a helpful instructional practice for reading new words.	1.67
2	4	Fluency and expression are necessary components of reading that indicate good comprehension.	1.83
3	21	Formal instruction in reading is necessary to ensure the adequate development of all skills used in reading.	2.00
4	12	Paying close attention to punctuation marks is necessary to understand story content.	2.04
5	7	It is good practice to allow children to edit what is written into their own dialect when learning to read.	2.08

**Table 3:** Means of the last five items of the Theoretical Orientation to Reading Profile

Ranking	Item	Content	Mean
24	13	It is a sign of an ineffective reader when words and phrases are repeated.	3.18
25	27	It is not necessary to introduce new words before they appear in the reading text.	3.25
26	10	It is good practice to correct a child as soon as an oral reading mistake is made.	3.60
27	23	Children's initial encounters with print should focus on meaning, not on exact graphic representation.	3.83
28	15	When coming to a word that is unknown, the reader should be encouraged to guess based on meaning and continue.	4.29

The whole‐language perspective defined by DeFord (1985) focusses on learners' acquisition of reading through reading an extensive amount of quality literature. Given that English is a foreign language in China, there is no natural environment for the acquisition of English language; therefore, it is understandable that no participants held a whole‐language perspective. Fifty‐eight participants (22%) identified their theoretical orientation to reading instruction as the decoding perspective—that is, a systematic and synthetic approach to teaching reading by decoding letters before studying word unit and comprehension. With the inclusion of phonics in the English textbooks of primary schools, teachers are encouraged by the curriculum to teach phonics when teaching English reading. Therefore, a considerable number of teachers (*N* = 204) chose a skills perspective, focussing on the instruction of vocabulary and their letter–sound correspondence in the context of the text they are studying, which was evident in the ranking of the items.

It was not surprising that all case study teachers held a skills perspective. However, even with three teachers, there were variations in how they perceived teaching reading. Xiaomei and Qingqing had TORP scores (68 and 69, respectively) that were more towards the decoding end of the continuum, whereas Lili's score (89) was in the middle range of the skills perspective. The case studies provided insight into possible influences on their perspectives including the differing content of TPD programmes. Xiaomei mentioned that phonics instruction was highlighted in various TPD programmes that she had recently attended. Therefore, it was understandable that she had TORP scores more towards the decoding end of the continuum. In contrast, Lili, who had attended TPD programmes emphasizing the analysis of texts with a discourse perspective, prior to TPD focussing on phonics instruction, approached texts with a more holistic view.

### 3.2 Effects of demographic variables on the TORP

Because the TORP results were categorized into two groups: a decoding perspective and a skills perspective, chi‐square tests were computed to generate inferential statistics. Results in Table 4 indicated that gender differences had a statistically marginally significant influence on the participants' TORP: *χ* ^2^(1, *N* = 262) = 3.147, *p = .*076. Female participants were more likely to have a skills‐based perspective, whereas male participants were more likely to have a decoding perspective. However, statistically, educational background, educational institutions, school locations, highest qualification obtained, and teaching experience did not have significant impact on their TORP (educational background: *χ* ^2^(1, *N* = 262) = 0.300, *p* = .584; educational institutions: *χ* ^2^(1, *N* = 262) = 0.564, *p* = .453; school locations: *χ* ^2^(2, *N* = 262) = 3.550, *p* = .169; highest qualification obtained: *χ* ^2^(3, *N* = 262) = 1.232, *p* = .745; and teaching experience: *χ* ^2^(4, *N* = 261) = 1.444, *p* = .837).

**Table 4:** Effects of demographic variables on the TORP

Variables	Levels	TORP	
		Decoding	Skills
Gender	Male	7 (12%)	11 (5%)
	Female	51 (88%)	193 (95%)
Educational background	English major	49 (84%)	178 (87%)
	Other majors	9 (16%)	26 (13%)
Educational institution	Teachers college and normal universities	46 (79%)	152 (75%)
	Other universities	12 (21%)	52 (25%)
School location	Cities	36 (62%)	110 (54%)
	Towns	18 (31%)	60 (29%)
	Rural areas	4 (7%)	34 (17%)
Highest qualification	College diploma	1 (2%)	4 (2%)
	Bachelor's degree	54 (93%)	184 (90%)
	Master's degree	3 (5%)	12 (6%)
	Other (less than 3‐year programme)	0 (0%)	4 (2%)
Years of teaching	1–3	9 (16%)	27 (13%)
	4–6	8 (14%)	35 (17%)
	7–18	33 (57%)	113 (56%)
	19–30	7 (12%)	27 (13%)
	30+Missing 1	1 (1%)	1 (1%)

*Note.* Numbers in parentheses indicate column percentages.

Abbreviation: TORP, Theoretical Orientation to Reading Profile.

To sum up, the survey teachers responded as expected, as the 2011 Curriculum Standards suggested a skills‐based approach for reading instruction. This is consistent with the researchers' observation of primary school teachers' general approach to English reading instruction, based on the researchers' long‐term involvement in English teacher education in Zhejiang Province.

### 3.3 Teachers' knowledge of basic language constructs related to language acquisition

The SBLCRLA comprises two parts: teachers' self‐efficacy beliefs about reading instruction and teachers' knowledge. Scores of perceived abilities were calculated by using a four‐point Likert scale, in which 1 = *minimal*; 2 = *moderate*; 3 = *very good*; and 4 = *expert.* Altogether, there was a maximum of 28 points of perceived abilities. The results indicated that the scores of perceived abilities ranged from 8 to 28 points: *M* = 17.206, *SD* = 3.59. Twenty‐six per cent of participants scored lower than 15 points, 13.3% scored higher than 20 points, and 1.5% achieved the full score, indicating that the majority of teachers rated their ability to teach reading as moderate. The participants rated their ability to teach vocabulary to be the highest (*M* = 2.77, *SD* = 0.683), followed by their ability to teach phonics (*M* = 2.71, *SD* = 0.710). Their ability to teach reading to struggling readers received the lowest score (*M* = 1.99, *SD* = 0.745).

The second part of the SBLCRLA was used to investigate the EFL teachers' metalinguistic knowledge and skills required to teach reading effectively. To calculate the scores, each correct answer was assigned one point, with a total possible maximum score of 55. The results indicated that the participants' scores ranged from 1 to 51 points: *M* = 30.61, *SD* = 10.918. In total, 7.6% of the participants scored lower than 10 points, and 17.1% scored higher than 40 points.

The survey items were grouped into two categories—knowledge and skill—in accordance with the validation study by Binks‐Cantrell et al. (2012). Knowledge items were concerned with “explicit knowledge of a term or concept,” whereas skill items were concerned with “implicit ability to perform task” (Binks‐Cantrell et al., 2012, p. 165). Table 5 presents the breakdown of survey items and examples, according to the two categories in the adapted survey. The survey aimed to explore EFL teachers' phonological‐, phonemic‐, morphological‐, and phonics‐based knowledge and skills (Zhao et al., 2015).

**Table 5:** Breakdown of survey items in knowledge and skill categories

Category	Example	Item numbers
Knowledge	A phoneme refers to: • a single letter • a single speech sound • a single unit of meaning • a grapheme • no idea	Q9, Q11, Q14, Q19, Q20, Q21, Q22, Q23, Q24, Q25, Q26
Skill	If “tife” was a word, the letter “i” would probably sound like the “i” in • if • beautiful • find • ceiling • sing • no idea	Q10, Q12a–g, Q13, Q15, Q16, Q17, Q18‐1‐a–e, Q18‐2‐a–e, Q27a–f

Table 6 presents the results of the breakdown of the survey items. The statistical analysis demonstrated that the majority of participants knew the term phoneme (*M* = 0.69, *SD* = 0.461), yet very few knew the meaning of phonemic awareness (*M* = 0.294, *SD* = 0.456), and even fewer knew the meaning of phonological awareness (*M* = 0.168, *SD* = 0.375). In terms of counting speech sounds, the highest score was observed for the word “ship,” with three phonemes (*M* = 0.92, *SD* = 0.266), which was also the highest score among all items of the survey. Following was the word “moon,” with three phonemes (*M* = 0.866, *SD* = 0.341), whereas “box” was the most difficult word, with four phonemes (*M* = 0.30, *SD* = 0.458).

**Table 6:** Breakdown of scores of knowledge and skills for all participants

Knowledge and skills	Full score	Minimum	Maximum	Mean	*SD*
Phonemic knowledge	3	0	3	1.28	0.85
Phonemic skill	10	0	10	6.94	2.33
Phonological knowledge	1	0	1	0.17	0.37
Phonological skill	5	0	5	3.29	1.75
Phonics knowledge	7	0	7	3.68	1.41
Phonics skill	1	0	1	0.90	0.30
Morphological knowledge	1	0	1	0.70	0.46
Morphological skill	27	0	26	13.70	6.90

*Note. N* = 262.

The teachers were more competent in the implicit skill of phonics than they were at describing terminology and explicit rules. For example, a high score (*M* = 0.90, *SD* = 0.305) was observed in finding a matching word that had a letter “i” that was pronounced the same as the long vowel /ai/ in a pseudo word “tife.” In contrast, a comparatively low score (*M* = 0.47, *SD* = 0.500) was observed in distinguishing “consonant blend” from “consonant digraph,” “silent consonant,” and “diphthong.”

Comparatively speaking, the participants performed better on the phonological‐based items, especially the syllable counting items, than on the morphological‐based knowledge items, as shown in Table 7. The items required the participants to identify the number of syllables and number of morphemes for the same sets of words. In total, 79.4% of participants could identify that “heaven” comprises two syllables, but only 45.8% understood that it consists of only one morpheme. On average, there was a 65.9% correct response rate for syllable counting, whereas the correct response rate for morpheme counting was much lower, at 47.02%.

**Table 7:** Percentage of participants correctly identifying numbers of syllables and morphemes

Words	Number of syllables (%)	Number of morphemes (%)
Disassemble	55	42
Heaven	79.4	45.8
Bookkeeper	63	47.7
Frogs	54.6	41.6
Teacher	77.5	58
	65.9 (mean)	47.02 (mean)

To sum up, the average score of the teachers' knowledge was low, indicating general insufficiencies in such knowledge among the 262 survey participants. The data collected from the case study teachers also indicated a lack of such a knowledge base. This lack of teacher knowledge also aligned with the findings of a number of studies, both internationally (Moats, 1994; Washburn, Joshi, & Binks‐Cantrell, 2011) and in Guangdong Province, China (Zhao et al., 2015).

Data from interviews showed that none of the case study teachers identified their own knowledge as a key component in effective teaching. They reported that, because they could speak and read English, they had sufficient skills to teach the language. However, Cunningham, Zibulsky, and Callahan (2009) have asserted that “being a skilled reader is not a sufficient condition for being a skilled reading teacher” (p. 504). These teachers' knowledge gaps might be due to the lack of explicit education on the components of English language acquisition in teacher education programmes in China, as reflected in the courses reported by the case study teachers. Zhao et al. (2015) also asserted that it is very likely that the teacher educators who designed the programmes themselves lacked adequate knowledge of this area and overlooked the importance of this knowledge for effective reading instruction, which echoes “The Peter Effect,” whereby one cannot give what one does not possess (Applegate & Applegate, 2004). This was also confirmed by several studies demonstrating teacher educators' lack of such requisite knowledge as a barrier to providing adequate instruction for preservice teachers in language structure (Joshi et al., 2009; Moats, 2014).

### 3.4 Effects of demographic variables on scores of knowledge and perceived abilities

Mann–Whitney tests were computed to examine the influence of external variables on the participants' scores of knowledge and perceived abilities because the data did not meet the assumption of normality. As displayed in Table 8, gender differences did not have a significant influence (*U* = 2,027.5, *p* = .587, *r* = .03) on scores of knowledge but had a significant influence (*U* = 1,585.5, *p* = .048, *r* = .12) on scores of perceived abilities, with a small effect size. These results indicated that male participants were more confident in their abilities to teach reading, whereas female participants were more reserved when discussing their abilities to instruct English reading. These results could be caused by limitations to the self‐report measures, indicating that the participants may not have reported their abilities accurately. Alternatively, they may be gender differences in the perception of teaching abilities.

**Table 8:** Effects of demographic variables on scores of perceived abilities and knowledge

Variables	Levels	Perceived abilities (mean)	Knowledge (mean)
Gender	Male (*N* = 18)	165.42	122.14
	Female (*N* = 244)	129	132.19
Educational background	English major (*N* = 227)	131.41	133.56
	Other majors (*N* = 35)	132.11	118.14
Educational institution	Teachers college and normal universities (*N* = 198)	138.60	132.85
	Other universities (*N* = 64)	109.55	127.32
School location	Cities (*N* = 146)	156.06	134.95
	Towns (*N* = 78)	98.76	124.28
	Rural areas (*N* = 38)	104.33	133.09
Highest qualification	College diploma (*N* = 5)	124.80	114.50
	Bachelor's degree (*N* = 238)	126.81	135.91
	Master's degree (*N* = 15)	183.30	96.87
	Other (less than 3‐year programme; *N* = 4)	224.50	20.13
Years of teaching	1–3 (*N* = 36)	138.81	104.40
	4–6 (*N* = 43)	136.40	132.70
	7–18 (*N* = 146)	123.43	134.37
	19–30 (*N* = 34)	149.25	142.46
	30+ (*N* = 2)Missing (*N* = 1)	116.75	132.50

Educational background had no significant influence on scores of knowledge (*U* = 3,505, *p* = .262, *r* = .07) or perceived abilities (*U* = 3,951, *p* = .959, *r* = .003) nor did teaching experience on scores of knowledge, (*χ* ^2^(4, *N* = 261) = 5.574, *p* = .233) or perceived abilities (*χ* ^2^(4, *N* = 261) = 4.174, *p* = .383). Educational institutions did not appear to have a significant influence on scores of knowledge (*U* = 6,068.5, *p* = .611, *r* = .03) but had a significant influence on scores of perceived abilities, with a small effect size (*U* = 4,931, *p* = .007, *r* = .17). Participants who graduated from teachers college and normal universities thought they had higher abilities than did participants who graduated from other universities. Given that the participants who graduated from teachers college and normal universities had received professional preservice teacher training, whereas the participants graduating from other universities usually had not received such type of training, it is highly likely that this might be why the former participants perceived themselves as holding higher abilities than did the latter participants. It was interesting that participants who held a bachelor's degree generally had higher scores for knowledge, yet this was not the case for those who held a master's degree. This could be attributed to the undergraduate degree being in an area not related to teaching, which suggests that the level of educational qualification was not a significant factor in their knowledge.

Level of education had a significant influence on both scores of knowledge (*χ* ^2^(3, *N* = 262) = 12.851, *p* = .005) and scores of perceived abilities (*χ* ^2^(3, *N* = 262) = 14.13, *p* = .003). To identify the significant differences among four types of degrees, multiple testing was performed. Given that six comparisons were made, the desired significant *p* value should be lower than .0083 after Bonferroni correction was performed.

Post hoc Mann–Whitney tests indicated that participants with a master's degree had higher perceptions of their abilities (*M* = 178.53) to teach English reading than did participants with a bachelor's degree (*M* = 123.75), after Bonferroni correction (*U* = 1,012, *p* = .005, *r* = .18), suggesting a small‐to‐medium effect size. There was a significant difference in the knowledge scores between the participants with a bachelor's degree (*M* = 123.27) and participants who had studied less than 3‐year programme degrees (*M* = 16.25), after Bonferroni correction (*U* = 55, *p* = .002, *r* = .20), suggesting a small‐to‐medium effect size. It is interesting to note that the group of “other” (degrees less than a 3‐year programme) achieved the highest (*M* = 224.50) among the four groups with regard to perceived teaching abilities yet scored lowest (*M* = 20.13) in the knowledge survey. These participants might have received their first degree from an open university, adult college, secondary specialized school, technical school, teacher training school, or correspondence school and later upgraded their degrees. Thus, they usually had not received professional English education, and their overall quality was not high (Xiong & Ma, 2013). Their perceptions did not seem to match their performance.

A close examination of the participants in the “other” group indicated that their years of teaching ranged from 2 to 7 years, which could explain the overrating of their teaching abilities. Previous research has indicated that teachers who are in the educational system for a longer time might be more cautious about rating their teaching abilities, whereas novice teachers tend to overrate their teaching abilities (Zhao et al., 2015). In contrast to the findings of Washburn et al. (2011) that teachers tend to overestimate their teaching abilities based on their knowledge of basic language constructs, one interpretation of the data is that teachers might also overestimate their teaching abilities because they are unaware of their lack of knowledge. This echoes the Dunning–Kruger effect, wherein individuals of low ability mistakenly rate themselves higher than average because they are unaware of what they do not know (Kruger & Dunning, 1999). However, because of the very small size of the sample, this result could also derive from pure chance. Therefore, it should be emphasized that any inference drawn from this sample should not be extrapolated to a larger population.

### 3.5 Correlation analysis between scores of knowledge and perceived abilities

Teachers' perceptions of their own efficacy in instruction play an important role in their belief system, which influences their students' achievements (Caprara, Barbaranelli, Steca, & Malone, 2006). Teachers' knowledge has also been correlated with students' academic achievements (Carlisle, Correnti, Phelps, & Zeng, 2009; Lane et al., 2008; McCutchen, Green, Abbott, & Sanders, 2009), with research confirming the connection between teachers' knowledge and effective reading instruction (Piasta et al., 2009; Snow & Griffin, 2007). Given that there is a significant influence of both teachers' self‐efficacy beliefs and knowledge in relation to reading instruction, the current research explored if there was a correlation between teachers' beliefs and knowledge. A Spearman's rank‐order correlation was computed to determine the relationship between the 262 participants' scores of knowledge and perceived abilities. The results indicated that scores of knowledge and perceived abilities were not statistically correlated (*r* = .040, *p* = .517).

A possible explanation for this result is that the participants did not report their abilities accurately because of a universal human trait due to the Dunning–Kruger effect. This is consistent with the findings of Main (2014) and Sharp, Brandt, Tuft, and Jay (2016). It also supports Bandura's (1977) self‐efficacy theory indicating that self‐efficacy does not rely on actual knowledge but is based on a perceived belief in one's ability to succeed.

Although the survey results indicated no correlation between teachers' self‐efficacy beliefs and knowledge of basic language constructs related to reading instruction, data triangulation from the case study teachers did indicate that teachers' beliefs about effective reading instruction were grounded in their relevant knowledge, even though it sometimes appeared tacit and insufficient. Further, the case study component of the research identified a complex relationship between teachers' knowledge and beliefs, as documented in the literature (Lin, 2013). The teachers' knowledge and beliefs established a dynamic web of influence that shaped their instructional decisions and practices. This was clearly demonstrated by the case study teachers' decision to code shift from L2 to L1 during classroom instruction, although they knew they were expected to use more English in the class to provide students with more exposure to the target language. For example, they would rely on L1:

- when language items were difficult, and using English would make explanations more complicated;
- when they were unable to use metalanguage to express difficult language points;
- when they needed to maintain classroom discipline; and
- when they encouraged students to engage in activities.

In Instance 1, their belief about comprehensible input took priority over their belief in exposure to the target language. In Instance 2, their belief about strategic compensation overrode that of exposure to the target language. In Instance 3, they believed that efficient communication in classroom management was more important. In Instance 4, their belief about the significance of building rapport by using L1 overrode that of exposure to English language. The teachers' strategic code shift of L1 and L2 in reading instruction illustrates the existence of multiple belief systems competing for actions and the adjustment of beliefs at a particular teaching moment constrained by a particular context. This is consistent with the assertion by Pajares (1992, p. 325) that knowledge and beliefs are “inextricably intertwined.” It also supports the literature's discussion on the interdependence of knowledge and beliefs (e.g., Hashweh, 2005).

### 3.6 Influence of teachers' knowledge and beliefs on their instructional practices

On the basis of the classroom observations and interviews, it was indeed found to be true that the three teachers were teaching from a skills perspective, and explicit phonics instruction was not provided. In addition, multiple sources of data consistently indicated that teachers lacked metalinguistic knowledge; for example, Xiaomei appeared unable to use metalanguage to provide reading instruction. Her teaching of how to form the simple past tense of verbs was problematic because she did not communicate to the class the concept of verb inflections, either in English or in Chinese. Nor could she explain to the class when to add the suffix “‐d” or “‐ed” to a verb to create the past form. This could have resulted from her insufficient knowledge of basic language constructs related to literacy acquisition, as indicated by the survey data. Previous research has suggested that an implicit knowledge of how language works does not necessarily translate into teachers' ability to teach children about the rules of the English language (Stark, Snow, Eadie, & Goldfeld, 2016). A lack of metalinguistic knowledge has been found to hinder teachers from providing explicit instruction of language structure in the classroom (Fielding‐Barnsley, 2010; Washburn et al., 2011) and from communication of professional knowledge (Moats, 2009). Overall, the case study teachers' reading instruction tended to converge with their beliefs, which confirmed that ESL teachers generally teach according to their theoretical beliefs (Johnson, 1992); however, the results also acknowledged the complexity of this relationship. For example, Lili seemed to hold a more holistic view towards teaching reading, yet, in practice, her reading instruction also highlighted explicit instruction of discreet skills and language items.

This research has identified a number of factors that influenced the incongruency between teachers' beliefs and instructional practices, including curriculum requirements, school context, and teacher education. Curriculum requirements—such as the need to cover the prescribed teaching content and the overwhelming examination‐oriented culture—resulted in a lack of time for extracurricular reading, causing conflicts and clashes between the teachers' beliefs and practices. These factors were also evident across different situations (Farrell & Bennis, 2013; Zhang & Liu, 2011). In this regard, perhaps the strongest influence is the examination‐oriented culture, where teachers are expected to “teach to the test” and prepare students to achieve a good result in the final assessment. This poses a challenge for teachers to engage in a range of reading assessment, integrating formative assessment with summative assessment.

School context—including the school location, socio‐economic status of the school community and parents, students' general English level, pressure for competitive teaching, daily routines, and school leadership—also had an influence on the incongruency between teachers' beliefs and practices. Some of these contextual factors and constraints have also been documented in the literature (Larenas et al., 2015). Located in low socio‐economic communities, both case study schools were less privileged than primary schools in larger cities in terms of resources, including financial funding, teacher development opportunities, and community and parental support. The case study teachers expressed concern over the limited resources, such as funding for buying extracurricular reading materials, and lack of parental support to foster students' home reading habits. They also reported that the pressure from competitive teaching for good examination outcomes and daily routines hampered them in implementing certain reading activities.

Teacher education—including preservice teacher preparation courses and in‐service TPD programmes—had a strong influence on the degree of consistency between teachers' beliefs and practices, which has also been documented in the literature (Deal & White, 2006; Main, 2014). The case study teachers identified in‐service TPD programmes as exerting more influence. This could be because the courses in the preservice teacher education did not specifically enhance the cultivation of primary school English teachers. A follow‐up discussion with the case study teachers revealed that the courses they attended at university aimed primarily to cultivate English teachers for secondary schools. The curriculum attached importance to the training of student teachers' general English linguistic knowledge and skills and general teaching approaches instead of cultivating English teachers' specific professional knowledge and skills. This is not uncommon in normal universities specializing in cultivating English teachers for primary and secondary education in contemporary China and was also identified by Zou's (2009) research. Zou purported that simply combining courses related to general English linguistic knowledge and skills with general teacher pedagogical courses cannot generate courses that accommodate the training of English teachers' specific professional knowledge and skills. Further, Zhao et al. (2015) observed that, although courses such as English phonology are tied to the specific training of English teachers' expertise in many English teacher preparation programmes, they are typically aimed at enhancing student teachers' own reading abilities, rather than focussing on how to integrate such knowledge and skills into instruction for students.

## 4 CONCLUSIONS

The findings of this research add to a growing body of literature on teachers' knowledge and beliefs about effective EFL reading instruction. It revealed that most of the participating teachers favoured teaching reading from a skills perspective rather than phonics or whole language perspectives. Another important finding is that there was general insufficient knowledge of language constructs related to reading instruction. Although the survey results indicated that there was no correlation between teachers' self‐efficacy beliefs and knowledge of the basic language constructs, a complex relationship did exist between teachers' knowledge and beliefs and that the dynamic interplay between the network of teachers' knowledge, beliefs, and instructional practices was mediated by the Chinese EFL contextual factors. This contributes additional evidence suggesting the need to enhance teachers' awareness of the significant impact of their own knowledge and beliefs on their practices. It is recommended that policy development, practices of EFL reading instruction, and preservice and in‐service EFL teacher development programmes in China consider incorporating teachers' knowledge and beliefs to improve the effectiveness of English language pedagogy.

This research has a few limitations. First, although the uneven gender distribution of the survey participants is representative of the current composition of primary school English teachers in China, caution should be taken when drawing implications from some of the survey findings because of the very small sample of male participants. Second, the approach to data collection is subject to limitations. Consideration should be given to the way in which knowledge was measured. The SBLCRLA mainly involved phonemic, phonological, phonics, and morphological knowledge and skills, with 27 items. Thus, this might cover only a fraction of teacher knowledge in relation to reading instruction. In addition, self‐report measures are criticized as unreliable (Onafowora, 2005) and could have created inaccuracies in the survey. It should also be noted that such findings may not be generalizable even to other parts of China. Future studies might include more male participants in the survey and case studies. In addition, more work is required to assess teachers' knowledge related to reading instruction in the other Chinese EFL contexts through observation of teacher practices.

## INITIAL OBSERVATION INTERVIEW GUIDE

How do you teach English reading?

What do you think effective EFL reading instruction involves?

(Let the participants talk from their own experience first and then develop questions based on their talks.)

- What materials would facilitate effective reading instruction?
- What instructional procedures do you think we should have to be able to teach reading effectively?
- What knowledge of English language is necessary to teach reading effectively?
- What learning activities should be incorporated into effective reading instruction?
- How do you think we should assess reading achievement?

Possible further questions:

- What materials do you use to facilitate effective reading instruction?
- Why do you use these materials?
- What are the benefits and limitations in using the materials?
- If you were the policymaker, what changes you would like to make to improve instructional practices?

## POST‐OBSERVATION INTERVIEW GUIDE

- Would you please briefly describe what you planned and implemented for the lesson? What are your objectives? Instructional procedures? Materials? Learning activities?
- Please look at the list of the observed practices I made. Is there anything missing?
- What is important in terms of effective English reading instruction from your perspective?
- Would you please tell me the reasons for using the practices I observed in this lesson? On what conditions do you do so? Have you ever made a different attempt? What happened?
- How did you learn to teach it that way? What has influenced the way you teach?
